# Fibroepithelial Lesion Spectrum: A Case Report Documenting a Possible Transformation to a Malignant Phyllodes Tumor

**DOI:** 10.7759/cureus.38252

**Published:** 2023-04-28

**Authors:** Amanda Felsen, Catherine Maldjian, Laura Hodges, Anjuli Gupta, Susan Fineberg

**Affiliations:** 1 Radiology, Albert Einstein College of Medicine, Bronx, USA; 2 Medicine, Montefiore New Rochelle Hospital, New Rochelle, USA; 3 Radiology, Montefiore Medical Center, Bronx, USA; 4 Surgery, Montefiore Medical Center, Bronx, USA; 5 Surgery, Albert Einstein College of Medicine, Bronx, USA; 6 Pathology, Montefiore Medical Center, Bronx, USA; 7 Pathology, Albert Einstein College of Medicine, Bronx, USA

**Keywords:** breast, phyllodes, fibroadenoma, transformation, fibroepithelial lesion

## Abstract

We discuss the radiological-pathological aspects of a rare case of transformation of a presumed fibroadenoma (FA) to a malignant phyllodes tumor (PT) and review the literature. Phyllodes tumors often show heterogeneous histologic features with some areas indistinguishable on core needle biopsy. A core biopsy is often a small representation of a larger lesion. As such, a complete excisional biopsy is often needed for a definitive pathologic diagnosis. Careful clinical and imaging correlation and follow-up are necessary, even in a benign fibroepithelial lesion (FEL).

## Introduction

Fibroepithelial lesions (FELs) range from benign to malignant neoplasms that present with varying degrees of stromal proliferation compared to epithelial components [1]. Fibroepithelial lesions of the breast include both fibroadenomas (FAs) and phyllodes tumors (PTs). Fibroadenomas are most common, have a benign nature, and are often found in young women. Diagnosed histologically, they typically present ductal components surrounded by loose fibrous tissue and have been described to have intracanalicular and/or pericanalicular growth patterns [2]. On mammography, they are often small in size, rounded, with well-circumscribed margins, and appear hypoechoic on ultrasound [3]. Pure fibroadenomas are usually surgically excised if they are growing, symptomatic, or greater than 3 cm in size.

Phyllodes tumors comprise <0.5% of all breast malignancies [4]. They typically occur in older women with an average age of 45 years and have malignant potential with possible local invasion and rarely hematogenous metastasis [4]. Phyllodes tumors are graded as benign, borderline, and malignant based on stromal features, including stromal overgrowth, and other contributing factors like nuclear atypia and the presence of mitoses. Diagnosis is confirmed by core needle biopsy (CNB), and phyllodes tumors are histologically characterized by intracanalicular growth patterns with leaf-life projections and some mesenchymal components [2]. On imaging, phyllodes tumors appear larger with irregular borders and microlobulations. By ultrasound imaging, they appear heterogeneous with increased vascularity [3]. Previous studies have shown that older age, rapidly growing lesions, and >3 cm tumor size are more likely associated with borderline and malignant phyllodes tumors and are more often indicated by ultrasound findings of irregular masses with poorly circumscribed or undefined borders. Wide surgical excision with 1 cm margins without nodal evaluation is considered the standard of care for phyllodes tumors as they tend to behave like sarcomas with hematogenous spread most commonly to the lung and bone [1]. Tumors that are histopathologically classified as malignant are at high risk for metastasis and may require mastectomy if clear margins cannot be achieved with wide local excision. There may be a higher local recurrence rate with close or positive margins, but final margin widths remain an area of discussion as distant metastases are more common in malignant phyllodes [5]. Chemotherapy can be considered for systemic treatment in cases of malignant phyllodes tumors with a high metastatic potential [6,7]. Various studies have shown decreased rates of local recurrence with adjuvant radiation therapy, but current guidelines only recommend consideration of adjuvant radiation with malignant phyllodes [8].

The literature has shown that it is very difficult to distinguish between phyllodes tumors and other benign or malignant tumors of the breast based on imaging and pathology. We document an unusual case of transformation of a presumed fibroadenoma to malignant phyllodes. We discuss the literature surrounding the fibroadenoma-phyllodes relationship, addressing imaging features and limitations of pathology.

## Case presentation

**Figure 1:**
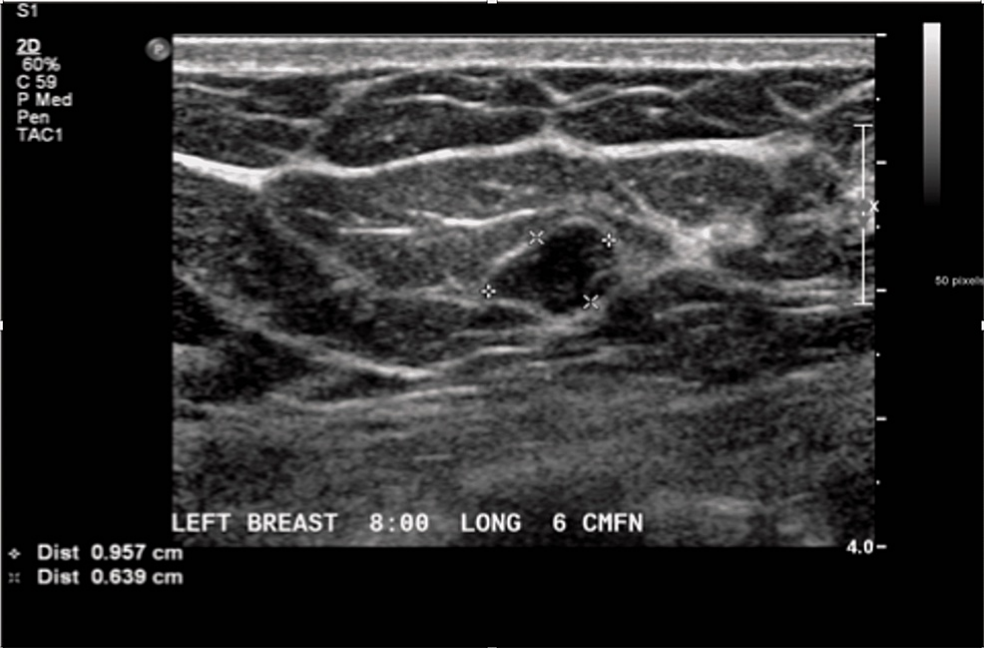
Breast ultrasound demonstrates a well-defined ovoid hypoechoic 1 cm nodule.

A post-menopausal female in her sixth decade presented with a new, nonpalpable left breast nodule on a screening mammogram. Ultrasound showed a 1 cm smooth hypoechoic nodule (Figure 1). As this was a new nodule in a post-menopausal patient compared to one year and four months prior to her presentation, when the patient had a normal outside mammogram, CNB was performed [4]. Ultrasound-guided biopsy yielded a fibroepithelial lesion with mildly increased stromal cellularity without significant cellular atypia or mitoses. The patient was nulliparous and had never been pregnant, had undergone menopause, and had no known history of breast cancer. The mass was followed with serial ultrasounds to screen for interval growth. It was stable in size over 15 months, but the patient was lost to follow-up.

**Figure 2:**
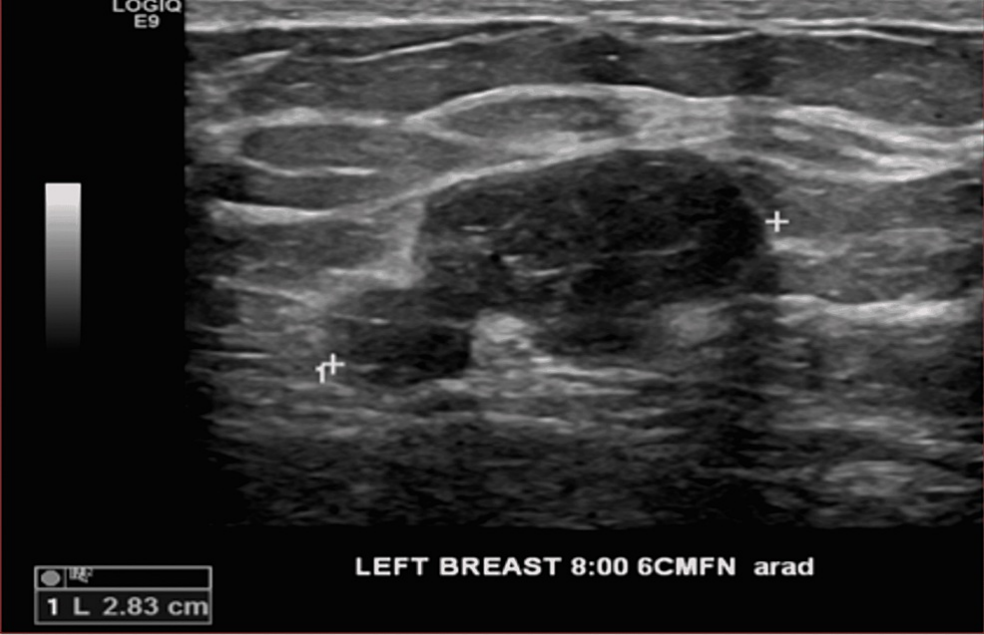
Ultrasound 32 months later demonstrates a hypoechoic 2.8 cm mass that has increased in size, with heterogeneity and lobulations.

**Figure 3:**
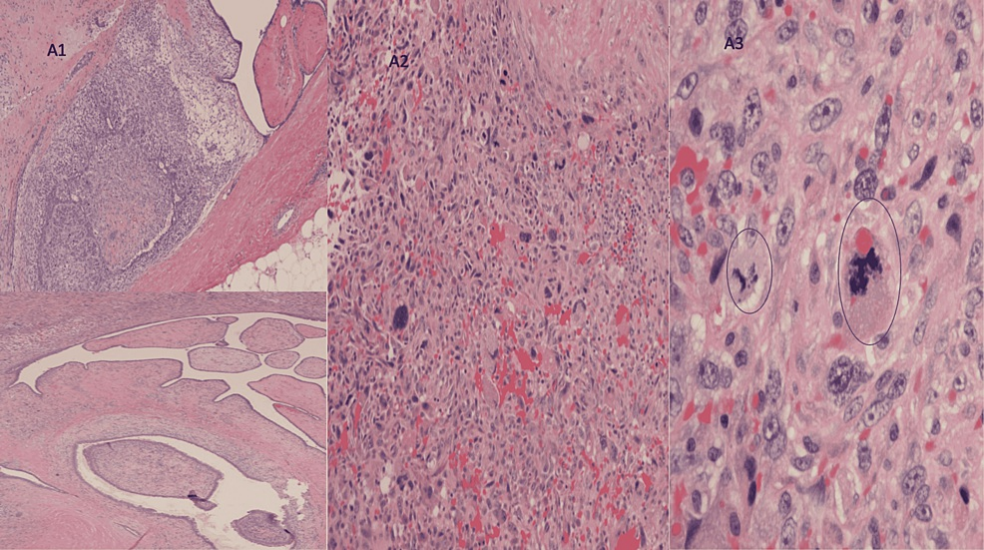
Malignant phyllodes tumor showing (A1) leaf-like growth and variable cellularity including high stromal cellularity, (A2) high stromal cellularity and marked nuclear atypia, (A3) mitotic figures including atypical mitotic figures.

Seventeen months later (32 months after initial presentation), the nodule grew to 2.6 × 1.7 × 2.8 cm, changing from smooth and homogeneous to lobulated and heterogeneous (Figure 2). The patient underwent a repeat percutaneous core biopsy of the mass. Pathology was reported as fibroepithelial lesion concerning for borderline phyllodes tumor with 40 mitoses per 10 high power field and mild to moderate cytologic atypia, with definitive diagnosis requiring excision. The patient was referred to a breast surgeon and underwent left breast segmental resection with 1 cm margins except for a close 5 mm anterior margin abutting the skin. Final pathology reported a 28 mm phyllodes tumor with foci of high stromal cellularity, marked stromal atypia, mitotic figures up to 15-20 per 10 high power field, stromal overgrowth, and foci of tumor infiltration into adipose tissue, all pathologic features of malignant phyllodes tumor (Figure 3). Immunostaining was positive for p53 in stromal cells, and the c-kit was largely negative.

The patient underwent a staging CT scan of the chest to rule out lung metastases, which was negative. The final American Joint Committee on Cancer (AJCC) staging was pT1NxMx. The patient was referred to radiation oncology for consultation and consideration of adjuvant radiation therapy to decrease her risk of local recurrence. She was treated with six weeks of 60 Gy adjuvant radiation therapy. Her clinical breast exam was normal after adjuvant therapy, and follow-up breast imaging was benign. The patient remains clinically and radiographically with no evidence of disease for >1 year and is followed by her oncology team every six months with annual CT chest and laboratory exams.

## Discussion

Fibroepithelial lesions (FELs) encompass a spectrum of histologic findings, ranging from fibroadenoma to malignant phyllodes that can appear similar radiographically. Cellular fibroadenoma, benign phyllodes, and borderline phyllodes lie along the continuum that progresses from fibroadenoma to malignant phyllodes. Cellular FAs reside on the cusp of PT within a grey area along this continuum. Cellular fibroadenoma often requires excision based on size or inability to distinguish CNB from more aggressive fibroepithelial lesions, such as PT. The difference in surgical management is that benign FELs (FAs are included in this) are typically managed surgically by enucleation only, whereas only FELs with malignant potential (i.e., atypical and malignant PTs) require local excision. Increased stromal cellularity, stromal mitosis, stromal overgrowth, fragmentation, nuclear pleomorphism, and infiltrative borders are associated with the upgrade at surgery [9]. These were largely absent at CNB for our patient, showing only mildly increased stromal cellularity without malignant features. A two-year follow-up of indeterminate FEL where 43 were excised, of which 13 were benign PT, with no borderline or malignant cases [10]. Thus, two years of stable findings on imaging is reasonable for excluding malignancy. Our case failed to show stability during the follow-up period.

PT occurs at an older age than FA, and in one study, the median age of occurrence for PT and FA was reported to be 39 and 23, respectively. Hence our patient’s age would favor PT [11]. Lesion margins, boundaries, and echo patterns sonographically show no significant difference between benign, borderline, and malignant phyllodes [5]. Lobulation and heterogeneous echotexture are sonographic features that distinguish phyllodes from FA [12]. The smooth margins and homogeneous echotexture at presentation in our case favored FA and supported the CNB diagnosis of benign FEL. However, subsequent lobulated margins and heterogeneous echotexture favor PT. This transition on ultrasound suggests FA with transformation to malignant phyllodes. Alternatively, PT heterogeneity can cause sampling error, and in a small lesion, this heterogeneity might not be appreciated on ultrasound. A prior investigation documents two cases of benign PT at surgery among a group of 83 fibroadenomas by CNB demonstrating growth [13]. About 60% discordance between the CNB pathology and excision pathology was seen in another study, attributed to insufficient sample size and tumor heterogeneity [14].

Apart from sampling error, breast pathologists demonstrate low agreement, achieving 100% agreement in only two out of 21 cases [15]. The agreement rate improves when FA, cellular FA, and benign PT are combined into one category and borderline/malignant PT are combined into another category, wherein breast pathologists achieve agreement 53% of the time [15]. In another investigation of 69 cases of equivocal FEL, 23% confidently diagnosed as FA on CNB were PT on excision. Low concordance, between 9% and 30%, is reported amongst pathologists in the categorization of FELs [16].

While cases of transformation of FA to PT are rare, identical somatic MED12 mutations and clonal relatedness analysis in FA and PT in the same patient support the hypothesis that a subset of FA are clonal, neoplastic, and can progress to benign/malignant PT [17,18].

A report of a patient with a 9 mm nodule showed FA at CNB with five years of mammographic stability. However, in the sixth year, the mass grew to 4 × 3 cm and surgery revealed malignant PT [19].

Another report of a 40-year-old woman revealed fibroadenoma at core biopsy, which doubled in size in the fourth year of follow-up and was diagnosed as malignant PT at excision [20]. At year three, ultrasound showed heterogeneity and a less well-defined appearance, similar to our case, though not excised at that point due to size stability. This suggests that even absent growth, sonographic changes may warrant excision.

## Conclusions

In summary, we document a rare occurrence of transition from a histologically benign, imaging stable FEL to a malignant PT, highlighting some of the challenges in the management and diagnosis of these lesions. As seen in our case and reviewed in the literature, an initial biopsy can be limiting due to the heterogenous nature of PTs and their ability to resemble benign FAs. Relative stable imaging characteristics for significant periods of time before evidence of transformation to a more malignant-appearing nodule confounds radiologists. While some evidence has shown that interval growth and older age should trigger consideration for excision, which is required for definitive diagnosis, there is no definitive mechanism to distinguish between these two fibroepithelial lesions before significant evidence of change on imaging leads to a high clinical suspicion, especially in the setting of a previous CNB that showed a benign lesion. Our case highlights the importance in closely analyzing these cases, especially in older populations with some interval growth, to ensure the correct diagnosis and treatment of these patients.
